# Effect of Bacterial Infection on the Edibility of Aquatic Products: The Case of Crayfish (*Procambarus clarkii*) Infected With *Citrobacter freundii*

**DOI:** 10.3389/fmicb.2021.722037

**Published:** 2021-09-29

**Authors:** Xiaoli Huang, Minghao Li, Jincheng Wang, Lili Ji, Yi Geng, Yangping Ou, Shiyong Yang, Lizi Yin, Liangyu Li, Defang Chen

**Affiliations:** ^1^Department of Aquaculture, College of Animal Science and Technology, Sichuan Agricultural University, Chengdu, China; ^2^Meat Processing Key Laboratory of Sichuan Province, Chengdu University, Chengdu, China; ^3^Department of Basic Veterinary, College of Veterinary Medicine, Sichuan Agricultural University, Chengdu, China; ^4^Chengdu Academy of Agriculture and Forestry Sciences, Chengdu, China

**Keywords:** *Citrobacter freundii*, crayfish, histopathological analysis, muscle quality, RNA-seq

## Abstract

Aquatic products are one of the world’s essential protein sources whose quality and safety are threatened by bacterial diseases. This study investigated the possible effects of bacterial infection on the main edible part, the muscle, in the case of crayfish infected with *Citrobacter freundii*. The histopathological analysis confirmed that crayfish was sensitive to *C. freundii* and muscle was one of the target organs. The transcriptome results showed impaired intercellular junctions, downregulation of actin expression, and inhibition of metabolic pathways. Furthermore, transcriptomic results suggest that *C. freundii* mainly affect muscle structure and nutrition. Subsequent validation experiments confirmed structural damage and nutrient loss in *C. freundii* infected crayfish muscle. Besides, the spoilage tests showed that *C. freundii* did not accelerate muscle spoilage and the bacteria had a limited impact on food safety. Therefore, although *C. freundii* may not be a specific spoilage bacterium, it still affects the edible taste and nutritional value of crayfish muscle. The findings of this study might contribute to further research on *C. freundii* infection and provide a warning about the adverse effects of bacterial infection on aquatic products.

## Introduction

With the increase in world’s population, the demand for food, especially high-protein animal-based foods, continues to expand (Sans and Combris, 2015; Kumar et al., 2017). Aquatic products have become one of the fastest-growing segments of global agriculture in recent decades due to their high-quality protein, high nutritional value, and low price (FAO). The red swamp crayfish (*Procambarus clarkii*) is widely farmed worldwide for its flavor, nutritional value, adaptability, and fast breeding (Holdich and David, 1993; Gong et al., 2008). However, with the increasing scale of the crayfish farming industry, the frequency of crayfish disease has increased (Longshaw, 2011; Shen et al., 2020). Diseases caused by many different pathogens have been reported in crayfish culture, including fungi such as *Batrachochytrium dendrobatidis* and *Aphanomyces astaci*, viruses as white spot syndrome virus (WSSV), bacteria, and *Spiroplasma* (Unestam and Weiss, 1970; Wang et al., 2005; Brannelly et al., 2015; Dong et al., 2016; Pace et al., 2016). Among infectious diseases, bacterial diseases are the most common in farming. Studies have shown that muscle is one of the target organs of many bacterial infections in aquatic animals, showing pathological signs such as congestion, hemorrhage, and necrosis after infection (Dong et al., 2017; Soares et al., 2019; Sun et al., 2020). In addition, reports on the nutritional loss of muscle caused by bacterial infections are also available (Berney and Berney-Meyer, 2017). Due to the profound potential impact of bacterial infection on muscle, the main edible part of crayfish, bacterial diseases cause severe economic losses to the crayfish farming industry. Therefore, it is imperative to study the effects of bacterial infections, especially newly reported ones, on crayfish muscle.

Recently, numerous reports have been published on crayfish infections caused by *Citrobacter freundii*, a bacterium typically found in the intestinal tract (Edgerton et al., 2002; Liu et al., 2020; Zhenbing et al., 2021). *C. freundii* is a Gram-negative, conditionally pathogenic bacterium belonging to the Enterobacteriaceae family and Citrobacter genus widely distributed in nature (Akoachere et al., 2009). Numerous studies have shown that *C. freundii* can infect newborns, horses, sheep and other mammals, causing meningitis and endocarditis (Guidi E. et al., 2016; Liu et al., 2018; Chen and Ji, 2019). In addition, aquatic animals are sensitive to *C. freundii*, and there have been reports of *C. freundii* infections in rainbow trout, silver catfish, eels, Chinese sturgeon, and crayfish (Aydin et al., 1997; Joh et al., 2013; Bandeira Junior et al., 2018; Liu et al., 2020; Yang et al., 2021). As an invertebrate, crayfish lacks the typical adaptive immunity and is more susceptible to mortality, inducing economic loss when infected with pathogenic bacteria (Cerenius et al., 2010). Meanwhile, the risk of food poisoning prevails for people who consume *C. freundii* infected crayfish (Pletz et al., 2018). Although *C. freundii* has posed a threat to crayfish farming and its food value, the effects of this bacterium on crayfish muscle remain relatively unknown.

In the present study, we investigated the molecular and physiological changes in the muscle of *C. freundii* infected crayfish. Our results provide a valuable theoretical basis for further study of the pathogenicity of *C. freundii* to crayfish and the effects of bacterial infection on the edibility of aquatic products.

## Materials and Methods

### Animals Preparation

Live crayfish (average weight 16.18 ± 1.10 g, random sex) were purchased from a local market in the Sichuan Province (China) and transported to the Fish Disease Research Center of Sichuan Agricultural University. Before the experiment, all crayfish were placed individually in a plastic box (19 cm × 12.5 cm × 7.5 cm) for 1 week to acclimate to the experimental conditions. The crayfish were fed commercial diets and dried mealworms (*Tenebrio molitor*) twice a day in the acclimatization period. The one-third of water was exchanged daily with fully aerated tap water. For experimental conditions, the temperature was maintained at 26°C, and the photoperiod by 14-h light and 10-h dark cycle. To confirm the absence of previous bacterial infection in experimental individuals, some of them were randomly tested using hepatopancreas and muscle. A total of 120 healthy crayfish with no injuries (intact appendages) and pattern behavior (walking and appendage movements) were randomly divided into two groups: control and infected groups.

### Experimental Infection

The experimental strain of *C. freundii* was isolated from dying crayfish by inoculating the solid medium with an inoculation loop, picking out the dominant colonies, and identifying with 16S rDNA sequencing. The strain was maintained as a frozen stock at −80°C in 50% (*v*/*v*) glycerol at Sichuan Agricultural University. After resuscitating the frozen stock culture, a single colony was transferred from the solid medium to 10 ml of LB broth medium by picking with an inoculating loop and shaking continuously at 28°C for 24 h. The strain was identified as *C. freundii* by DNA extraction, PCR amplification of the 16S rDNA fragment (forward primer: 5′- AGAGTTTGATCCTGGCTCAG -3′ and reverse primer: 5′- GGTTACCTGTTACGGACTT -3′) and Blast comparison of the NCBI database. The 1.5 ml of broth culture was centrifuged at 8,000 rpm for 10 min at 4°C to collect the sediment. The bacterial pellet was washed with sterile phosphate-buffered salt (PBS) and adjusted to a concentration of one-tenth of the LD_50_ (LD_50_ was derived from previous experiments as 2.7 × 10^5^ CFU/mL) and injected intramuscularly in the infected group, while the crayfish in the control group were injected with an equal volume of sterile PBS. The injected volume was 100 μL and the injection site was near the third abdominal segment of the crayfish. Fresh moribund crayfish from the infected group were collected for bacterial isolation. The isolated bacteria were identified using 16S rDNA sequencing. The experimental conditions were consistent with the period of acclimatization.

### Histological Examination

Ninety-six hours after infection, the muscle tissue of crayfish from the two groups was fixed with Davidson’s AFA fixative for at least 48 h for histological observation. For tissues containing crusts (muscle), decalcification was undertaken by immersion in the decalcification fluid for a further 24 h. Subsequently, all samples were trimmed and placed in embedding boxes, processed for paraffin embedding, sectioned at 5 μm, mounted and dried on slides, and stained with hematoxylin and eosin (H&E). Histopathological changes were observed under a microscope (Nikon, Tokyo, Japan).

The muscle wounds were evaluated after infection. The degree of hemorrhage, edema, deposits, hypertrophy, hyperplasia, atrophy, infiltration, and necrosis in organs was graded according to the scoring system proposed by Baums et al. (2013). The score for each crayfish was defined as the sum of the scores from eight different pathological changes divided by eight. Every change was assessed with a score (S) ranging from 0 to 6, depending on the degree and extent of the change: (0) unchanged; (2) mild occurrence; (4) moderate occurrence; and (6) severe occurrence (diffuse lesion).

### RNA-seq

#### RNA Extraction, Library Preparation and Illumina Sequencing

Ninety-six hours after bacterial infection, three replicates of muscle transcriptome samples per group were frozen in liquid nitrogen and stored at −80°C. Total RNA was extracted from the muscle tissue using TRIzol^®^ Reagent and genomic DNA was removed using DNase I (TaKara). Then, the quality and quantity of extracted RNA were determined by 2100 Bioanalyzer (Agilent Technologies, Inc., Santa Clara, CA, United States) and quantified using the ND-2000 (NanoDrop Thermo Fisher Scientific, Wilmington, DE, United States). Only high-quality RNA samples (OD260/280 = 1.8∼2.2, OD260/230 ≥ 2.0, RIN ≥ 8.0, 28S:18S ≥ 1.0, >1 μg) were used to construct the sequencing library.

Muscle RNA-seq transcriptome libraries were prepared using Illumina TruSeq RNA sample preparation Kit (San Diego, CA, United States). Poly(A) mRNA was purified from total RNA using oligo-dT-attached magnetic beads and then fragmented by fragmentation buffer. Taking these short fragments as templates, double-stranded cDNA was synthesized using a SuperScript double-stranded cDNA synthesis kit (Invitrogen, CA, United States) with random hexamer primers (Illumina). Then the synthesized cDNA was subjected to end-repair, phosphorylation and “A” base addition according to Illumina’s library construction protocol. Libraries were size selected for 200–300 bp cDNA target fragments on 2% Low Range Ultra Agarose followed by PCR amplification using Phusion DNA polymerase (New England Biolabs, Boston, MA, United States) for 15 PCR cycles. After quantified by TBS380, two RNA-seq libraries were sequenced in single lane on an Illumina HiSeq X Ten/NovaSeq 6000 sequencer (Illumina, San Diego, CA, United States) for 2 × 150 bp paired-end reads.

#### *De novo* Assembly and Annotation

The raw data were treated using SeqPrep and Sickle to remove the junk reads with adapters, >10% N base and low-quality scores (Q20) and the redundancies were filtered out. Then clean data from the samples were used for *de novo* assembly with Trinity software. All the assembled transcripts were searched against the NCBI Nr, GO, Pfam, Kyoto encyclopedia of genes and genomes (KEGG), COG, and Swiss-Prot databases using BLASTX to identify the proteins that had the highest sequence similarity with the given transcripts to retrieve their function annotations and a typical cut-off *E*-values less than 1.0 × 10^–5^ was set.

#### Differential Expression Analysis and Functional Enrichment

To quantify the expression level of transcripts, the transcripts per million reads (TPM) method using RSEM software was employed to convert mapped reads from alignments into the expected number of fragments per kilobase of transcript sequence per millions of base pairs sequenced (FPKM). The statistical analysis of differentially expressed genes (DEGs) was performed using the DESeq2 package with FDR < 0.05 and | log2FC| ≥1 as the default screening criteria. In addition, the functional enrichment analysis of the screened DEGs was performed by Goatools, and KOBAS with a Bonferroni-corrected *P*-value ≤ 0.05 determined the extent of DEG enrichment in different GO terms and metabolic pathways.

### Physical Parameters

The pH, shear force, and water-holding capacity of crayfish muscle in the two groups were measured 96 h after injection. For pH determination, we referred to GB 5009.237-2016, 1 g of muscle sample was homogenized in nine volumes of KCl buffer and suspended in a water bath at room temperature. The pH was determined using a pH meter calibrated at 26°C in pH 4.0 and 7.0 buffers.

Water-holding capacity is reflected by cooking loss, drip loss, and cooked meat percentage. Cooking loss was defined as the percentage of muscle weight loss after cooking at 70°C for 5 min compared with the initial weight. The drip loss was the weight loss ratio to the initial weight after hanging the muscle at 4°C for 24 h. The muscle weight ratio to the initial weight after cooking for 3 min and cooled for 20 min was taken as the cooked meat percentage (Wu et al., 2007).

Random selection of whole shrimps was used for shear force analysis which was the average value of peak force recordings across muscle block using a texture analyzer.

### Chemical Parameters

The muscles of crayfish 96 h after infection were collected, minced, and homogenized for the chemical analysis of moisture, proteins, amino acids, intramuscular fat (IMF), and fatty acids. For the detection of protein, fat, amino acid, and fatty acid, we refer to GB 5009.5-2016, GB 5009.6-2016, GB 5009.124-2016, and GB 5009.168-2016, respectively. The routine oven-drying method determined the moisture content. The Soxhlet extraction method measured the IMF content in duplicate, while the protein content was measured by the Kjeldahl method using a 6.25 factor to convert the nitrogen content into total protein (Horwitz et al., 1980; AOAC, 1990). Fat saponification and fatty acid methyl esterification were carried out in NaOH after fat extraction with ether, the fatty acid methyl ester content was obtained by gas chromatography and the different fatty acid contents were obtained by calculation. The hydrochloric acid hydrolysis of proteins was followed by the spectrophotometric determination of different amino acids by ion-exchange chromatography with ninhydrin columns.

### Spoilage Degree Analysis

After the injection experiment, the surviving crayfish were selected and executed. The muscles with shells were removed and placed in sealed bags to compare the spoilage rate of the two groups of muscles at different temperatures. For the excised muscle, the intestine was removed to avoid intestinal bacteria on the results. Muscle changes were recorded at 4°C for 2 days and at room temperature (26°C) for 1 day. The muscle in sealed bags was homogenized in distilled water. Total volatile basic nitrogen (TVB-N) was measured by the colorimetric method. The pH value of the homogenate supernatant was measured with a pH meter. An evaluation team of three experimenters evaluated each sample and determined the score for the degree of spoilage by selecting the appropriate descriptor from a list of five established parameters (Supplementary Table 1).

### Statistical Analysis

GraphPad Prism 8 was used for graphical representation and SPSS 26.0 software (SPSS Inc., Chicago, IL, United States) for statistical analysis. Data were expressed as mean ± standard deviation (SD). Statistical analyses were compared using the Student *t*-test, and statistical significance was defined as *P* < 0.05.

## Results

### Effect of *C. freundii* Infection

Our previous study indicated that *C. freundii* could infect crayfish and cause mortality (Feng et al., 2021). To examine whether *C. freundii* could cause muscle damage, crayfish were injected with a tenth of the LD50 of *C. freundii* in a subacute attack, while gross and histological changes in muscle were observed.

**FIGURE 1:**
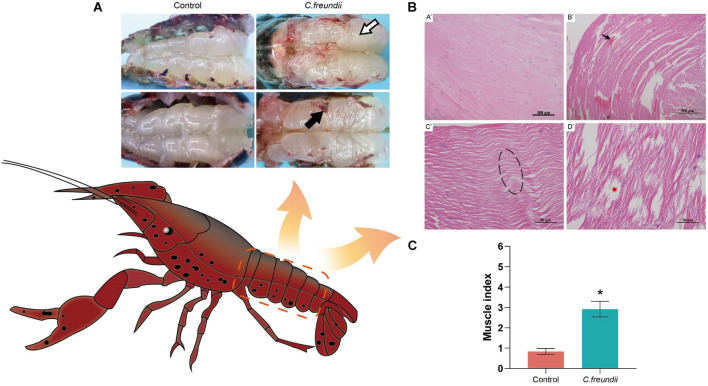
Gross pathology and histopathological observation in crayfish muscle. **(A)** Muscle gross lesions of crayfish after infection. Color is cloudy and dull (white arrow), and adhesion is strong (black arrow). **(B)** Pathological changes in crayfish muscle. **(A’)** Healthy hepatopancreatic histological characteristics; **(B’)** Myofibers degenerated and became indistinct, and tissues were sparse (black arrow); **(C’)** Muscle fibers were wavy (dotted box); **(D’)** Muscle bundles were dissolved and necrotic, and formed part of the cavity (red pentagram). **(C)** Muscle health status (organ index) of crayfish infected by *C. freundii* in different groups. *P < 0.05 represents a significant difference between the infected group (green bar) and the control group (red bars).

Some of the crayfish in the infected group died in the experimental infection and *C. freundii* was isolated from the hepatopancreas and muscle. The infected crayfish showed abnormal behavior such as anorexia and slow response. The muscles in the control group were white and elastic. However, cloudy and friable muscles were common in the infected crayfish group (Figure 1A). The H&E staining results showed that *C. freundii* infection caused severe muscular histological alterations in crayfish, showing marked disorganized muscle fibers, dissolved and necrotic muscle bundles, and cavity formation. No significant histological changes were observed in the muscles in the control group (Figure 1B). Note that granulocyte infiltration (inflammation), the typical histological change in pathogenic bacterial infections, was not observed in infected crayfish’s muscle organs. The pathological scores indicated significant pathological changes in the infected group muscles (Figure 1C).

### Sequencing Quality Assessment and Determination of DEGs

Three libraries were constructed with the RNAs from *C. freundii*-infected crayfish muscle to investigate the potential effect of *C. freundii* on muscle. Similarly, three libraries were constructed with the RNAs from the muscle of the control group. The six libraries were sequenced, and the data are summarized in Table 1. An average of 44,355,892 raw reads was obtained, 99.16% of which passed the quality filtering process. Further, 49,884 unigenes with N50 1926 bp were generated using the Trinity assembly program. Supplementary Figure 1 summarizes the length distributions of these assembled unigenes. This study matched clean reads from different samples to reference transcripts using RSEM^1^ for mapping analysis. The mapping rate of each sample was 83.25 ∼ 87.31%, meeting the requirements of RNA-seq analysis without a reference genome.

**TABLE 1:** Raw and clean reads generated for the muscle transcriptomes.

Sample	Raw reads	Clean reads	Clean read ratio	Q20 (%)*	Q30 (%)**	GC content (%)	Mapped ratio (%)
CM_1	45071814	44647058	0.990576	98.13	94.4	47.15	83.25
CM_2	46711218	46332616	0.9918948	98.18	94.5	50.74	85.12
CM_3	44947106	44627854	0.9928972	98.35	94.9	50.93	85.85
FM_1	41734940	41365852	0.9911564	98.22	94.67	49.33	85.93
FM_2	43676014	43279148	0.9909134	98.25	94.75	49.7	86.83
FM_3	43994260	43652026	0.9922209	98.26	94.68	50.84	87.31

*^∗^Percentage of bases with quality score ≥20 (bases with accuracy of 99%).*

*^∗∗^Percentage of bases with quality score ≥30 (bases with accuracy of 99.9%).*

To predict their possible functions, the assembled unigenes were BLAST searched against six public databases (Nr, Nt, Pfam, KOG/COG, Swiss-Prot, KEGG, and GO). A total of 17,319 unigenes were annotated. A Venn diagram shows the count of annotations in each database (Supplementary Figure 2) and presents 7,919 genes annotated together in all the databases. DESeq2 was used for differential expression analysis. DEGs were identified by the fold change in the gene expression level (log2| FC| > 1) and *P*-value (*P* < 0.05). Compared with the control group, the infected group exhibited 736 DEGs, of which 256 and 480 were upregulated and downregulated, respectively (Supplementary Figure 3).

### Kyoto Encyclopedia of Genes and Genomes Enrichment Analysis of the DEGs

**FIGURE 2:**
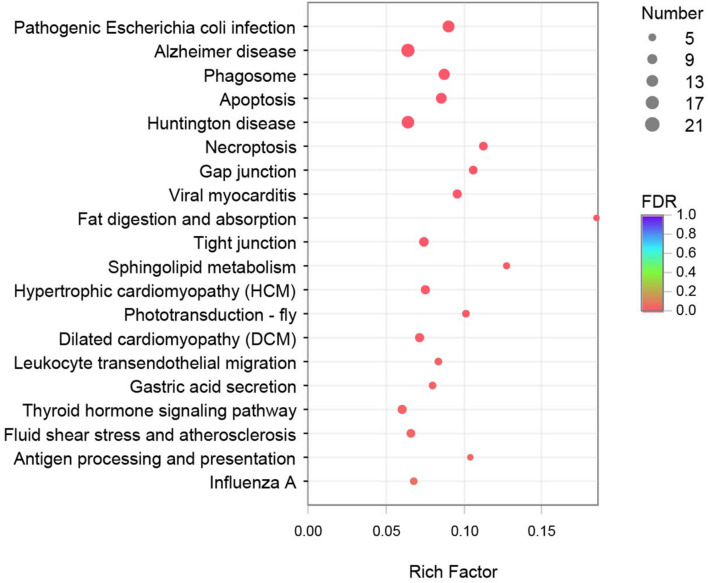
Top 20 pathways enriched in differentially expressed genes (DEGs) by KEGG. The color and size of the dots indicate FDR and DEG numbers, respectively.

KEGG enrichment analysis was performed to further understand the biological functions of the DEGs. All DEGs were mapped to each term in the KEGG database, and the number of genes in each term was calculated. The distribution of the top 20 KEGG terms significantly enriched by categories is shown in Figure 2. The KEGG analysis indicated that the main pathways underlying the differential expression in crayfish muscle infected with *C. freundii* included phagosome, apoptosis, necroptosis, gap junction, fat digestion and absorption, tight junction, sphingolipid metabolism, phototransduction, leukocyte transendothelial migration, gastric acid secretion, thyroid hormone signaling pathway, and antigen processing and presentation. Besides these immune and metabolism pathways, the terms related to various diseases and infections, including pathogenic *Escherichia coli* infection, Alzheimer’s disease, Huntington’s disease, viral myocarditis, hypertrophic cardiomyopathy (HCM), dilated cardiomyopathy (DCM), and fluid shear stress and atherosclerosis were also highly represented based on rich factor.

**FIGURE 3:**
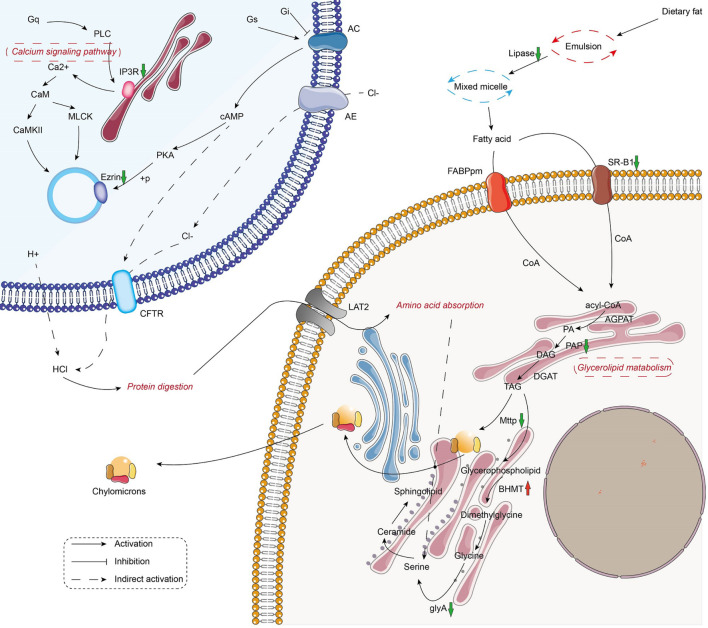
Mapping the metabolism-related gene regulation network in the muscle of infected crayfish based on transcriptome data. The red and green arrows indicate the up regulation and down regulation of DEGs, respectively.

**FIGURE 4:**
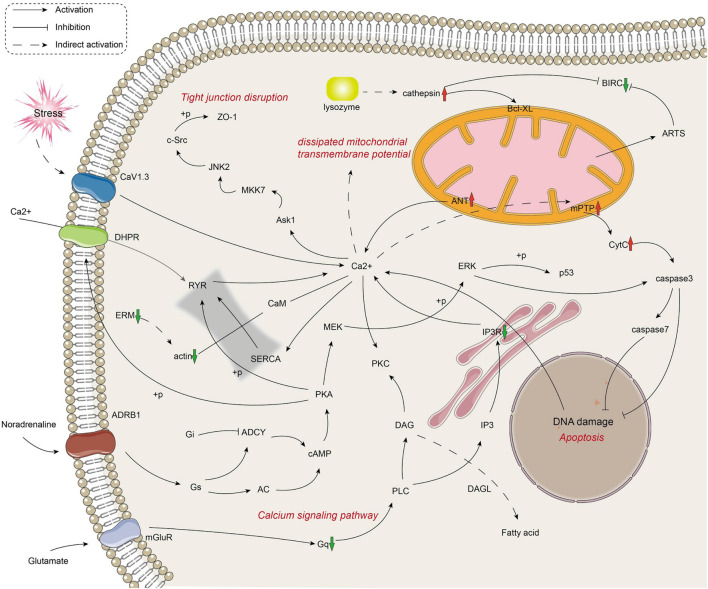
Mapping the immune-related gene regulation network in the muscle of infected crayfish based on transcriptome data. The red and green arrows indicate the up regulation and down regulation of DEGs, respectively.

The metabolic and immune gene regulatory network of infected crayfish was mapped according to the top 20 KEGG-enriched pathways (Figures 3, 4). The results showed that the calcium signaling pathway-dominated regulatory network appeared in infected crayfish muscle. The increased calcium ion content in the muscle cytoplasm of infected crayfish might lead to abnormal gastric acid secretion, disruption of tight junctions, impaired mitochondrial transmembrane potential and activation of apoptosis pathways. Furthermore, besides the apparent activation of pathways such as apoptosis and necrosis, we observed down regulated expression of *actin* genes associated with muscle composition and inhibition of metabolic pathways. Based on the mapping of gene regulatory networks and the results of pathological observations, we hypothesized that *C. freundii* infection of crayfish might cause alterations in the physical structure and nutrient composition of muscle.

### Bacterial Infection Affected the Physical Qualities of Muscle

**FIGURE 5:**
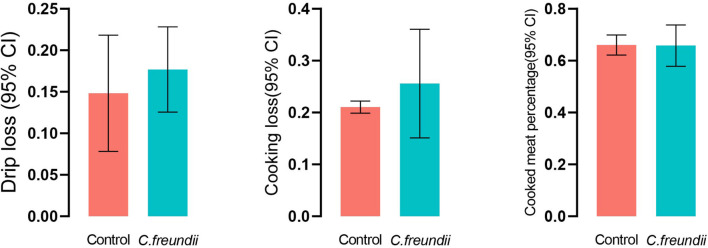
Comparison pf the water-holding capacity of the two groups.

The physical structure and nutrient composition of crayfish muscle were determined to test our hypothesis based on the transcriptome results. Table 2 and Figure 5 show the physical parameters of the distinct groups of muscles. We found that *C. freundii* infection affected multiple physical characteristics of muscle. The pH is an important parameter influencing the technological and sensory qualities of muscle, mainly influenced by the content of glycogen and amino acid breakdown products, and reflecting the effects of external stress on the muscle (Gonzalez-Rivas et al., 2020). In the present study, the pH value in crayfish with *C. freundii* infection was significantly improved compared with that in the control group, but still in the normal range. In addition, the water-holding capacity of meat reflects the integrity of the physical structure of muscle, influencing the weight of the final product and consumer acceptance. Water loss can be due to evaporation, drip loss, or cooking (den Hertog-Meischke et al., 1997). We found that despite no significant difference between the two groups, drip loss and cooking loss were relatively higher in the muscles in the infected group than in the control group. Furthermore, the *C. freundii* infection also tended to decrease the cooked meat percentage. Another aspect of the effect of *C. freundii* on the physical structure of muscle is reflected by the higher SF values in the injected group compared with the control group, meaning a decrease in muscle tenderness.

**TABLE 2:** Descriptive statistics of carcass traits and common meat quality traits in two groups of crayfish.

Parameters	Control		*C. freundii*		*P*-value
	Mean	C.V^1^	Mean	C.V	
Body weight (g)	16.30 (1.50)**^2^**	9.20	16.05 (0.51)	3.18	0.628
Muscle weight (g)	1.93 (0.48)	24.8	1.80 (0.31)	17.2	0.483
Moisture (g/100 g)	81.4 (1.03)	1.27	81.2 (0.38)	0.47	0.767
pH	6.50 (0.00)	0	7.04 (0.01)	0.14	7.83E-8
Shear force (g)	273		350		

*^1^Coefficient of variation.*

*^2^Standard deviation.*

### Effect of *C. freundii* Infection on Nutritional Composition of Muscle

**FIGURE 6:**
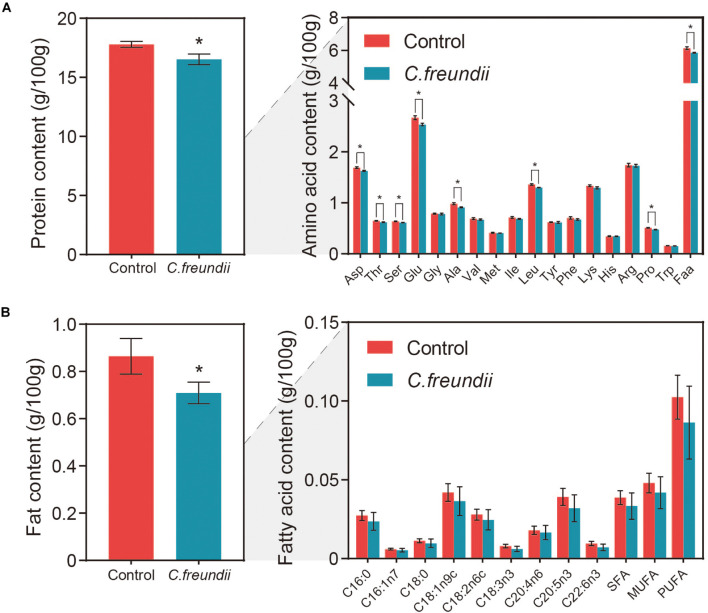
Content of nutrients in crayfish muscle. **(A)** Protein and amino acid contents in crayfish muscle. Faa, Flavor amino acids, mainly including glutamic acid, aspartic acid, alanine, and glycine. **(B)** Fat and fatty acid contents in crayfish muscle. SFA, Saturated fatty acids, mainly including C16:0 and C18:0. MUFA, Monounsaturated fatty acids, mainly including C16:1n7 and C18:1n9c. PUFA, Polyunsaturated fatty acids, mainly including C18:2n6c, C18:3n3, C20:4n6, C20:5n3, and C22:6n3 *P < 0.05.

Aquatic products mainly provide consumers with protein and fat, two of the three primary nutrients (carbohydrate, fats, and proteins) (Hosomi et al., 2012; Oehlenschläger, 2012). However, some factors, including bacterial infection, affect the nutritional composition (Rohmer et al., 2011). We assessed the degree of variation in protein content, fat content, 17 amino acids, and nine measurable fatty acid compositions between the two groups to evaluate the effect of *C. freundii* on the on the nutritional value of crayfish muscle (Figure 6). Remarkably, muscle protein and fat contents were significantly lower in the infected group than in the control group. Specifically, the contents of all 17 amino acids in muscle were reduced after infection, with a significant reduction in aspartic acid, threonine, serine, glutamic acid, alanine, leucine, and proline. Moreover, the flavor amino acid content was also reduced by *C. freundii* infection. Besides, regarding the fatty acid composition of the muscles in different groups, polyunsaturated fatty acids (PUFA), saturated fatty acids (SFA), and monounsaturated fatty acids (MUFA) showed a decreasing trend after infection. Similarly, the content of all nine measurable fatty acids also decreased. The results demonstrated that the contents of two main nutrients in muscle declined after *C. freundii* infection.

### *Citrobacter freundii* Infection Did Not Accelerate Muscle Spoilage

**FIGURE 7:**
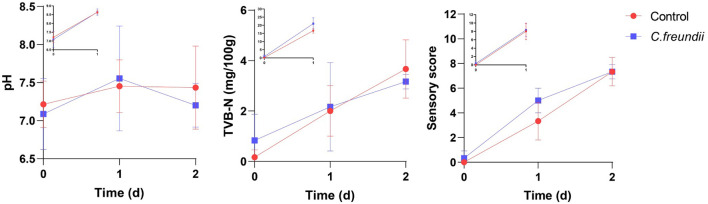
Changes in pH, TVB-N, and sensory scores at different temperatures in the two groups. The main axis represents the change in indicators at 4°C; the top left shows the change in indicators at room temperature.

We tested the rate of muscle spoilage after infection to clarify whether *C. freundii* affected food safety. For most aquatic products, the pH tended to decrease during the initial stages of spoilage because glycolysis produces lactic acid, and ATP and creatine phosphate were broken down into acidic substances. As proteins and amino acids are broken down to produce alkaline substances such as amines, the pH increased again (Bhadra et al., 2015). Total volatile base nitrogen is an important indicator of the spoilage of aquatic products, as it includes ammonia and low-level amines produced by enzymatic and bacterial protein breakdown during the spoilage process of animal food (Noseda et al., 2010). The dynamic changes in TVB-N and pH are shown in Figure 7. The TVB-N concentration in both infected and control groups did not exceed 20 mg/100 g at 4°C, with no significant difference between the two groups. In contrast, the TVB-N concentration in the two groups was much higher during storage at room temperature than at 4°C after 1 day, approaching 20 mg/100 g. However, no significant difference between the two groups was observed. Meanwhile, the pH value in the two groups was initially about 7.1. During storage at room temperature, the pH values in both groups increased substantially, reaching values of approximately 8.6. In contrast, the pH value in the two groups was fairly stable during 4°C storage, ranging from 6.7 to 7.9.

As shown in Figure 7, the freshness of the muscles in the two groups was excellent at the beginning. Also, the spoilage characteristics increased gradually over time, but with no significant difference in the scores between the two groups during 4°C storage. In contrast, the scores in the two groups at room temperature were not significantly different, but they were much higher than the scores at 4°C during the same time.

## Discussion

Bacterial diseases are among the most dangerous infectious diseases in aquaculture, and there are many reports of pathogens. However, most studies focused on immune organs with high blood flow (such as liver, spleen, and kidney) and pathogenic or immune response mechanisms (Gholamhosseini et al., 2018). Muscle is one of the potentially diseased organs that researchers can easily overlook when no apparent changes on the body surface are seen. In addition, even when focal lesions are found on the body surface or muscle tissue, management is often limited to observing pathological changes after sectioning (Huang et al., 2019). The effects of bacterial infections on muscle, especially on muscle nutrients, have not been studied enough. The impact of bacteria on the nutritional quality of aquatic products is equally significant compared with the economic damage caused by bacterial infections because aquatic products are one of the world’s primary nutritional sources.

Our results showed that *C. freundii* infection affected the arrangement of crayfish muscle fibers, with fractured muscle fibers, abnormal density, nucleus fixation, and red staining of the sarcoplasm. We examined 736 DEGs in infected crayfish muscle by transcriptome analysis to further explore the potential effect of *C. freundii* infection on crayfish muscle. Notably, the KEGG database enrichment pathway showed actin gene expression downregulation and inhibition of metabolic pathways in the gene regulatory network. Transcriptome-based validation showed that infected crayfish had reduced muscle water-holding capacity, disrupted muscle structure, and reduced protein and fat contents.

Actin is one of the most abundant proteins in any eukaryotic cell and is highly conserved. Besides being an indispensable component of the cytoskeleton, actin is involved in many motile activities such as intracellular vesicle transport, cytokinesis, and cell locomotion (Dominguez and Holmes, 2011). Studies have shown that many bacteria can directly attack actin or its manifold regulatory partners to disrupt cellular actin structures and reduce phagocytic activity in host cells. For example, *Clostridium botulinum* injects ADP-ribosyltransferase into host cells, which binds to actin and acts as a depolymerizer (Aktories et al., 1986); *Yersinia enterocolitica* produces the bacterial effector YopO which is activated after binding to G-actin, leading to the F-actin destruction and impairment of host phagocytosis (Trasak et al., 2007; Lee et al., 2015). In addition to the affected cytoskeletal component actin, we found that muscle cells’ intercellular junctional structures (tight junctions and gap junctions) were impaired. Both tight junctions and gap junctions play a role in mechanical connection and information transfer. The molecular composition, ultrastructure and function of tight junctions are regulated by physiological and pathological stimuli (Sawada, 2013; Dokladny et al., 2016). Besides signal transduction, the tight junction is also an essential part of innate immunity, because some tight junction proteins are receptors of viruses and participate in extracellular stimulation (Krug and Fromm, 2020). The dysregulation and loss of tight junction integrity contribute to disease progression (Buckley and Turner, 2018). It has been shown that the lipopolysaccharide component of the outer surface of the bacterial cell wall acts on intestinal epithelial cells with downregulation of tight junction protein, increased intestinal cell permeability and impaired intestinal barrier function (Guo et al., 2013, 2015). Gap junction proteins are structurally and functionally similar to tight junction proteins and exchange substances and signal transduction between adjacent cells; the disruption of their function can also lead to diseases (Nielsen et al., 2012). In this test, actin expression was downregulated and the tight junction and gap junction pathways were impaired. Subsequent tests confirmed that crayfish muscle had reduced water-holding capacity, suggesting that *C. freundii* infection could disrupt the structural integrity of muscle.

Transcriptome sequencing showed that the organism did not have an increased ability to utilize protein and fat; however, the muscle nutritional quality of crayfish infected with *C. freundii* was reduced. This suggested that the loss of nutrients was more likely to be caused by bacteria rather than organismal regulation. Bacterial pathogens can use their hosts as a rich source of nutrients to support their own survival and replication. Research has shown that the metabolism of bacteria within the host is a key determinant of the virulence and persistence of infection (Best and Abu Kwaik, 2019). Because bacteria require high levels of nutrients, including sugars, amino acids, and lipids, to proliferate, they must obtain them from their hosts. For example, oxygenated mycoplasma acid molecules found on the surface of *Mycobacterium tuberculosis* can induce the conversion of lung macrophages into a phenotype known as “foamy macrophages,” where the bacteria can accumulate within the cells and use the host’s cholesterol and fatty acids to survive (Peyron et al., 2008; Gago et al., 2018). Microbial degradation enzyme systems are one of the mechanisms by which pathogenic bacteria make the most use of nutrients in the complex environment of the host. Bacteria can use bacterial enzymes, such as proteases or phospholipases, to degrade host macromolecules so as to extract essential nutrients (Passalacqua et al., 2016). *Vibrio cholerae* is capable of synthesizing NanH (a neuraminidase) to break down sialic acid and convert it into fructose-6-P that feeds into the glycolytic pathway (Jeong et al., 2009). *Francisella tularensis* secretes γ-glutamyl transpeptidase to use the amino acids in the cytoplasm as its main source of carbon and energy for proliferation (Alkhuder et al., 2009). On the other hand, bacteria can also manipulate host biological processes to obtain nutrients. The bacterial effector AnkB secreted by *Legionella pneumophila* is anchored to the cytosolic face of the Legionella-containing vacuole (LCV) membrane by a lipid modification, and triggers polyubiquitin tagging of proteins on the LCV surface. These ubiquitinated proteins are subsequently targeted to the 26S proteasome and rapidly degraded to generate a localized supply of amino acids (Al-Quadan and Kwaik, 2011; Wong et al., 2017). Based on the experimental results, it was more likely that *C. freundii* used microbial degradation enzymes to obtain nutrients from crayfish muscle.

In recent years, diarrhea and food poisoning cases in humans infected with *C. freundii* have been reported. We also studied changes in the rate of spoilage of crayfish muscle after infection to understand the impact of the bacterium on food safety. Microorganisms are the major cause of spoilage of most aquatic products (Gram and Huss, 1996). However, only a few members of the microbial community, the specific spoilage organisms (SSOs), participated in the spoilage process (Gram and Dalgaard, 2002). People who consume spoiled food can develop symptoms such as poisoning (Foo, 1975). In addition, nearly a quarter of the world’s food is wasted due to spoilage, so food spoilage caused by microorganisms is a global research topic (National Research Council Subcommittee on Microbiological Criteria., 1985). Crayfish muscle infected with *C. freundii* was not significantly different from that in the control group in terms of spoilage rate at 4°C versus room temperature, suggesting that *C. freundii* was not an SSO or inhibited by competition with other bacteria for nutrients (Gram et al., 2002). In addition, the rate of muscle spoilage was much lower at 4°C than at room temperature for the same time duration. For more perishable aquatic products, storage at low temperatures can better preserve the quality of the food.

## Data Availability Statement

The data presented in the study are deposited in the Sequence Read Archive (SRA) at the National Center for Biotechnology Information (NCBI), accession number PRJNA738709.

## Ethics Statement

The animal study was reviewed and approved by the Animal Care and Use Committee of Sichuan Agricultural University.

## Author Contributions

XH, ML, JW, LJ, and DC contributed to conception and design of the study and article writing and revision. ML wrote the first draft of the manuscript and performed the statistical analysis. YG, YO, SY, LY, and LL wrote sections of the manuscript. All authors contributed to manuscript revision, read, and approved the submitted version.

## Conflict of Interest

The authors declare that the research was conducted in the absence of any commercial or financial relationships that could be construed as a potential conflict of interest.

## Publisher’s Note

All claims expressed in this article are solely those of the authors and do not necessarily represent those of their affiliated organizations, or those of the publisher, the editors and the reviewers. Any product that may be evaluated in this article, or claim that may be made by its manufacturer, is not guaranteed or endorsed by the publisher.

## Footnotes

1
https://github.com/deweylab/RSEM/archive/v1.3.3.tar.gz
