# Supplementary material for: Effect of Bacterial Infection on the Edibility of Aquatic Products: The Case of Crayfish (Procambarus clarkii) Infected With Citrobacter freundii
Source: Front Microbiol. 2021 Sep 29;12:722037. doi: 10.3389/fmicb.2021.722037 (PMC8511708; doi:10.3389/fmicb.2021.722037)
Supplement: Supplementary file 1 [file Data_Sheet_1.PDF]

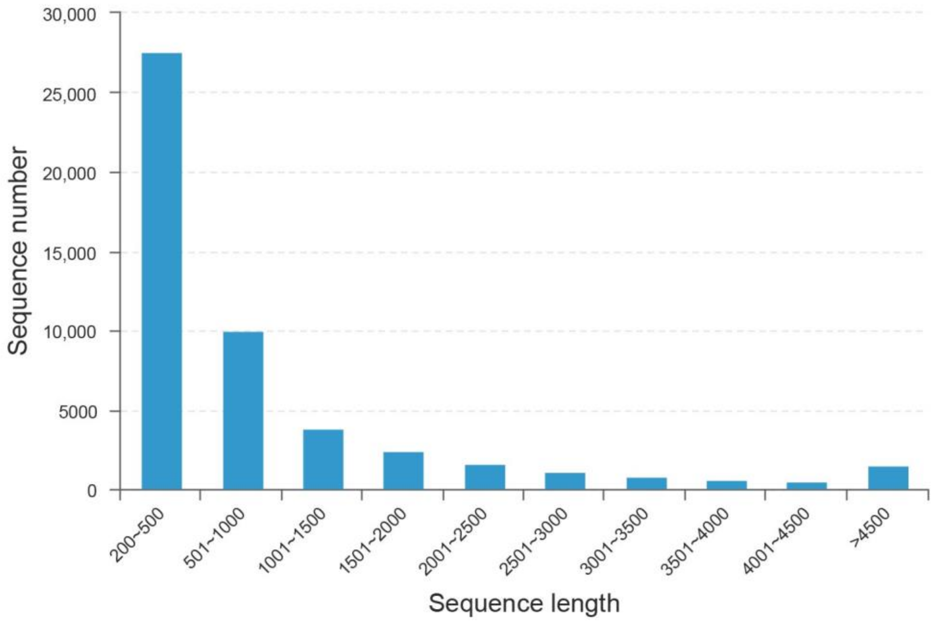

Supplementary Figure 1. Length distribution of unigenes in the muscle transcriptomes. The *X*-axis represents the length of unigenes, and the *Y*-axis stands for the number of unigenes.
