# Supplementary figures and images for: Effect of Bacterial Infection on the Edibility of Aquatic Products: The Case of Crayfish (Procambarus clarkii) Infected With Citrobacter freundii
Source: Front Microbiol. 2021 Sep 29;12:722037. doi: 10.3389/fmicb.2021.722037 (PMC8511708; doi:10.3389/fmicb.2021.722037)

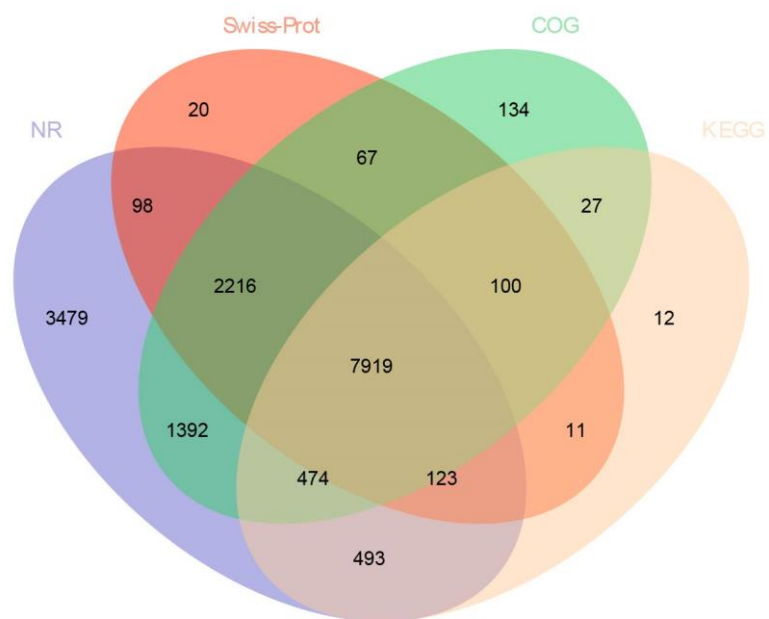

**Supplementary Figure 2.** Numbers of annotated unigenes in four public databases.

Supplement: Supplementary file 2 [file Data_Sheet_2.PDF]
