# Supplementary material for: Effect of Bacterial Infection on the Edibility of Aquatic Products: The Case of Crayfish (Procambarus clarkii) Infected With Citrobacter freundii
Source: Front Microbiol. 2021 Sep 29;12:722037. doi: 10.3389/fmicb.2021.722037 (PMC8511708; doi:10.3389/fmicb.2021.722037)
Supplement: Supplementary file 3 [file Data_Sheet_3.PDF]

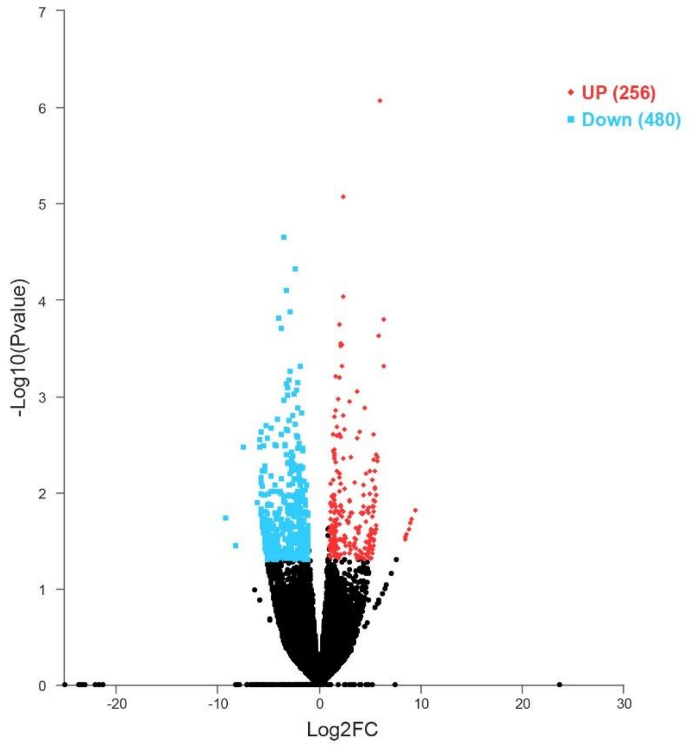

Supplementary Figure S3. Volcano plot showing the distributions of the differentially expressed genes (DEGs). Each dot represents a gene. Red and blue dots represent up- and down regulated DEGs, respectively
