# Supplementary material for: Effect of Bacterial Infection on the Edibility of Aquatic Products: The Case of Crayfish (Procambarus clarkii) Infected With Citrobacter freundii
Source: Front Microbiol. 2021 Sep 29;12:722037. doi: 10.3389/fmicb.2021.722037 (PMC8511708; doi:10.3389/fmicb.2021.722037)
Supplement: Supplementary file 4 [file Table_1.DOCX]

**Supplementary Table S1.**

Sensory evaluation table of spoilage degree

| **Sensory trait** | **Description** | **Score** |
| --- | --- | --- |
| Color/Luster | Glossy/White and transparent | 0 |
|  | Somewhat glossy/slightly transparent | 1 |
|  | Tarnish/Cloudy and opaque | 2 |
| Muscle texture | Tight muscle/Elastic | 0 |
|  | Loose/Sightly elastic | 1 |
|  | Poor elasticity/loose muscles | 2 |
|  | Slippery/Soft and paste | 3 |
| Characteristic flavor | Having shrimp characteristics with umami taste | 0 |
|  | Characteristic umami taste is lighter | 1 |
|  | Umami free | 2 |
| Spoilage odors | No fishy and rotten smell | 0 |
|  | Mild smell of ammonia, urea, and hydrogen sulfide | 1 |
|  | Moderate smell of ammonia, urea, and hydrogen sulfide | 2 |
|  | Strong smell of ammonia, urea and hydrogen sulfide | 3 |
| Connectivity | Muscle is tightly connected to the carapace | 0 |
|  | Muscle is relatively lax in connection with the carapace | 1 |
|  | Muscle and carapace detached | 2 |
| Index Total |  | 12 |
